# A Serratia marcescens brain abscesses in a preterm infant: An atypical presentation

**DOI:** 10.1016/j.radcr.2025.08.041

**Published:** 2025-09-16

**Authors:** Mohamed Sellouti, Salahiddine Saghir, Anass Ayad, Mehdi Bahouss, Rachid Abilkassem

**Affiliations:** a Neonatal Medicine and Intensive Care Unit, Mohammed V Military Teaching Hospital, Rabat, Morocco; b Faculty of Medicine and Pharmacy, Casablanca, Morocco

**Keywords:** Neonatal brain abscess, Prematurity, Serratia marcescens, Sepsis management

## Abstract

Brain abscesses represent a rare yet severe complication of bacterial sepsis in neonates, with Gram-negative bacteria being predominant etiological agents. While Serratia marcescens is an uncommon pathogen in neonatal sepsis and meningitis, it is recognized for its opportunistic nature and association with healthcare-associated infections. Despite advancements in antimicrobial therapies and diagnostic imaging, clinical outcomes in this population remain concerning, with high rates of mortality and long-term neurological sequelae. We present a case of a preterm neonate who developed multiple brain abscesses caused by Serratia marcescens

## Introduction

Brain abscesses in neonates are uncommon yet critical conditions, often linked to significant mortality and long-term complications [1,2]. Predisposing factors include underlying conditions such as prematurity, low birth weight, immune deficiencies, extended hospitalization in intensive care settings, mechanical ventilation, recurrent use of broad-spectrum antibiotics, and systemic infections [3]. Advances in diagnostic approaches—including advanced imaging techniques like ultrasound, computed tomography (CT), and magnetic resonance imaging (MRI)—coupled with microbiological analyses and more targeted antimicrobial therapies, have enhanced clinical management and outcomes [4].

This report details a neonatal case of multiple brain abscesses managed successfully through burr-hole aspiration and a prolonged antibiotic regimen. A review of the literature highlights key aspects of pathogenesis and contemporary strategies for managing such complex infections in this vulnerable population.

## Case presentation

A male infant was delivered via emergency cesarean section at 31 weeks’ gestation due to predelivery hemorrhage secondary to gestational hypertension. The neonate weighed 1,100 g at birth, with Apgar scores of 8 at 1 minute and 9 at 5 minutes. Postnatal respiratory distress necessitated continuous positive airway pressure (CPAP), which stabilized oxygen saturation above 90%, with transient tachypnea resolving within 6 hours.

Empirical intravenous benzylpenicillin and gentamicin were initiated but discontinued after 48 hours following negative blood cultures.

On day 4, the infant exhibited jaundice, tachypnea, feeding difficulties, and lethargy. Neurological assessment revealed physiological hypotonia without focal deficits, though a single seizure occurred on day 5, managed with phenobarbital. Laboratory findings included leukocytosis (38,000/µL) with neutrophilia (15,000/µL) and normal C-reactive protein (4.8 mg/L). Blood cultures identified Serratia marcescens, prompting escalation to meropenem (20 mg/kg every 6 hours) and amikacin (15 mg/kg daily). Cerebrospinal fluid (CSF) analysis and imaging (chest X-ray, arterial blood gas) were unremarkable.

The diffusimetric antibiogram was performed according to the EUCAST

(European Committee on Antimicrobial Susceptibility Testing) guide from 2010 using a Vitek2 Compact device (BioMerieux, Lyon, France). The results are shown in Table 1. On day 6 of life he was noted to have a left-sided grade II intraventricular haemorrhage (IVH). As he was asymptomatic at the time, this was managed conservatively with weekly monitoring.

**Table 1:** Antibiotic susceptibility of Serratia marcescens in our case.

Antimicrobial agent	Sensitivity
Ciprofloxacin	S
Meropenem	S
Amikacin	R
Amoxicillin	R
Ceftrixaone	S
Gentamycin	S
Piperacillin-tazobactam	I
Cefuroxime	R

At 30 days of life (corrected gestational age: 35 + 5 weeks), the infant experienced a severe apneic episode accompanied by generalized edema, delayed capillary refill (3 seconds), and a bulging anterior fontanelle. Suspecting recurrent sepsis, empirical therapy with cefotaxime (50 mg/kg every 12 hours) and amikacin (15 mg/kg daily) was initiated. Initial blood cultures returned sterile, and cerebrospinal fluid (CSF) analysis showed no pleocytosis or organisms on Gram stain, excluding meningitis. However, due to persistent clinical signs of sepsis—including irregular respiratory patterns—antibiotics were escalated after 48 hours to intravenous vancomycin (15 mg/kg every 12 hours) and meropenem (20 mg/kg every 12 hours). Subsequent blood cultures isolated *Serratia marcescens*, while repeat CSF studies remained unremarkable. Antimicrobial susceptibility testing, performed using EUCAST guidelines and the Vitek2 Compact system, guided further adjustments (results summarized in Table 2).

**Table 2:** Adjust antibiotic susceptibility of Serratia marcescens.

Antimicrobial agent	Sensitivity
Ciprofloxacin	S
Meropenem	R
Amikacin	R
Amoxicillin	R
Ceftrixaone	S
Gentamycin	S
Piperacillin-tazobactam	I
Cefuroxime	R

**Fig. 1:**
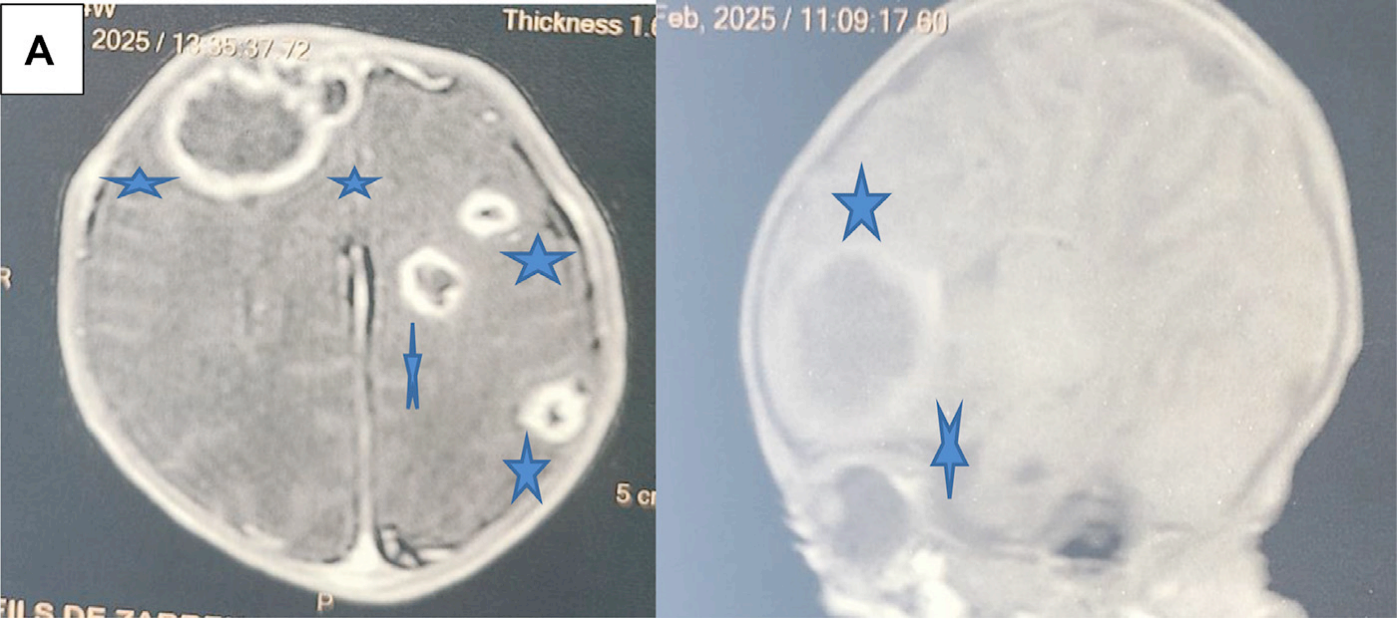
(A) Magnetic resonance imaging showed large cystic lesion in right frontal, parietal, temporal and occipital lobes. The largest lesion on the right side measured 2,3×1.3 cm and (B) Magnetic resonance imaging showed large cystic lesion in right frontal lobe.

Following the detection of *Serratia marcescens* recurrence, antimicrobial therapy was adjusted to intravenous ciprofloxacin (10–15 mg/kg/day) and gentamicin (5 mg/kg/day) for 7 days. A cranial MRI performed at 60 days of life (corrected gestational age: 39+5 weeks) demonstrated multiple contrast-enhancing lesions in the bilateral frontal and right parieto-occipital regions (Fig. 1A and B), confirming the diagnosis of multifocal brain abscesses. The multidisciplinary team opted for neurosurgical drainage combined with a tailored systemic antibiotic regimen comprising cefotaxime (150 mg/kg/day), ciprofloxacin, vancomycin (60 mg/kg/day). After completing an 8-week course of antibiotics, the infant was discharged in stable condition. At follow-up visits until 3 months of age, the patient exhibited no neurological deficits or recurrent infections, maintaining optimal clinical progress.

## Discussion

A brain abscess is a localized infection within the brain tissue, marked by the formation of a pus-filled cavity enclosed by a fibrous capsule. In neonates, this condition is exceptionally rare and typically arises as a secondary complication of bacterial meningitis or bloodstream infections. Pathogens can disseminate to the brain via hematogenous spread (through the bloodstream) or directly invade the cerebrospinal fluid (CSF) following traumatic injuries, surgical interventions, or infections such as otitis media. Gram-negative bacteria, including Proteus and Citrobacter species, are more frequently associated with neonatal brain abscesses due to their neuroinvasive potential. In contrast, Serratia marcescens is seldom identified as a causative agent, with only a limited number of cases documented in medical literature (2).

Serratia marcescens is ubiquitous and not a standard component of human fecal flora. Most infections are acquired exogenously [5]. A recent analysis of the causes of neonatal sepsis in developing countries cited the Serratia species as the fifth most common cause (0.5% for early neonatal sepsis and 0.3% for late neonatal sepsis) [6].

There have been outbreaks of Serratia marcescens in neonatal intensive care units described in the literature [[7], [8], [9], [10]]. Still, they often did not progress to the central nervous system or other complications. Usually, S. marcescens and cerebral infections are linked to cerebral instrumentations, such as neurosurgical procedures or lumbar punctures.

*Serratia marcescens* is an environmental bacterium not typically found in the human intestinal microbiota. Infections are primarily acquired through external sources, such as contaminated medical equipment or healthcare settings [5]. A recent epidemiological study in developing nations identified *Serratia* species as the fifth leading cause of neonatal sepsis, accounting for 0.5% of early-onset cases and 0.3% of late-onset cases [6]. While outbreaks of *S. marcescens* in neonatal intensive care units have been documented [[7], [8], [9], [10]], these incidents seldom progress to central nervous system involvement or severe systemic complications. Notably, cerebral infections linked to *S. marcescens* are most often associated with invasive medical interventions, such as neurosurgical procedures or repeated lumbar punctures.

The clinical manifestations of a brain abscess depend on variables such as the abscess's anatomical site, the pathogen's aggressiveness, and the host’s immunological competence. In neonates and infants, these infections frequently develop secondary to neonatal meningitis or systemic bloodstream infections [11]. Symptoms in this age group are often nonspecific, such as lethargy, feeding difficulties, or irritability, underscoring the need for heightened clinical vigilance. Routine laboratory tests, including complete blood counts, offer limited diagnostic utility. Cerebrospinal fluid (CSF) analysis may appear normal or mimic bacterial meningitis, with pleocytosis and elevated protein levels, but rarely provides definitive evidence [12]. Microbiological confirmation is challenging, as Gram stains and CSF cultures are typically negative, and blood cultures identify the pathogen in only approximately 10% of cases [13]. Consequently, neuroimaging modalities—particularly contrast-enhanced computed tomography (CT) and magnetic resonance imaging (MRI)—serve as the cornerstone for diagnosis, enabling precise localization and characterization of lesions [14,15].

The treatment of neonatal brain abscesses is guided by factors such as the lesion’s location, size, multiplicity, developmental stage, and the infant’s age and neurological stability [16]. Nonsurgical approaches are often prioritized to minimize procedural risks, particularly in fragile neonates susceptible to neurological impairments or hemorrhage associated with invasive interventions [18]. Antimicrobial therapy typically spans 6 to 8 weeks, with extended courses recommended for immunocompromised infants [17]. Despite these guidelines, optimal dosing and duration of antimicrobials for neonatal brain abscesses remain under-researched, highlighting a critical gap in evidence-based protocols [18].

Surgical management offers critical advantages, including obtaining microbiological specimens for precise diagnosis, debulking abscess volume to enhance antibiotic penetration, and enabling targeted antibiotic delivery (eg, intrathecal, intraventricular) in complex cases [19]. Despite these benefits, the long-term prognosis for neonatal brain abscess survivors remains concerning. Studies consistently report neurodevelopmental challenges, including cognitive deficits and academic difficulties, with many children exhibiting below-average IQ scores and impaired educational outcomes [2].

## Conclusion

Neonatal brain abscesses do not always follow meningitis, underscoring the importance of recognizing subtle or atypical clinical indicators. Beyond classical symptoms, clinicians must remain vigilant for unusual presentations such as irregular respiratory patterns, failure to sustain adequate oxygenation without respiratory support, or the absence of hallmark signs like fever or focal neurological deficits. In such scenarios, heightened clinical vigilance and a low threshold for neuroimaging are essential to ensure timely diagnosis and intervention.

## Patient consent

Written informed consent was obtained from the patient for publication of this case report.
